# Genetic Regulation of Immune Response in Dogs

**DOI:** 10.3390/genes16070764

**Published:** 2025-06-29

**Authors:** Pablo Barragán-Sánchez, María Teresa Balastegui, Pablo Jesús Marín-García, Lola Llobat

**Affiliations:** 1 Molecular Mechanisms of Zoonotic Diseases (MMOPS) Research Group, Departamento Producción y Sanidad Animal, Salud Pública y Ciencia y Tecnología de los Alimentos (PASASPTA), Facultad de Veterinaria, Universidad Cardenal Herrera-CEU, CEU Universities, 46113 Valencia, Spain; pablo.barragansanchez@alumnos.uchceu.es (P.B.-S.); maria.balastegui@uchceu.es (M.T.B.); 2 Departamento Producción y Sanidad Animal, Salud Pública y Ciencia y Tecnología de los Alimentos (PASASPTA), Facultad de Veterinaria, Universidad Cardenal Herrera-CEU, CEU Universities, 46113 Valencia, Spain; pablo.maringarcia@uchceu.es

**Keywords:** canine breed, cytokines, dog leukocyte antigen, immune system, epigenetic regulation, major histocompatibility complex, toll-like receptors

## Abstract

The mammalian immune system, including key components such as toll-like receptors (TLRs), lymphocytes, and cytokines, plays a vital role in defending against diseases. In dogs, genetic polymorphisms and epigenetic regulation of immune-related genes contribute to breed-specific differences in susceptibility or resistance to infectious, autoimmune, and inflammatory diseases. Cytokines, essential for immune cell differentiation and activation, exhibit variable expression among breeds due to genetic factors like single-nucleotide polymorphisms (SNPs) and miRNA regulation. This variability influences immune responses not only to infections but also to chronic inflammatory conditions and cancer, providing insights for improved diagnosis, treatment, and breeding. Selective breeding has further shaped diverse immune phenotypes across breeds, especially through genetic variations in the major histocompatibility complex (MHC) region, which affect vulnerability to immune-mediated and immunodeficiency disorders. Recent studies emphasize the role of specific miRNAs in modulating immune responses during parasitic and viral infections, opening new avenues for precision veterinary medicine and immunotherapy. This review highlights the genetic and epigenetic regulation of immune genes in dogs and explores their potential applications in advancing veterinary diagnostics, therapeutics, and breeding strategies to enhance canine health.

## 1. Introduction

The immune system is essential for host defense against pathogens and maintaining homeostasis in mammalian species. In dogs, like other mammals, immune responses are regulated by genetic factors that modulate the recognition of pathogens, signaling cascades of inflammation, cellular immunity, and antibody production, among others. Recent advances in canine genomics, including whole-genome sequencing and genome-wide association studies (GWAS), have found numerous loci associated with immune function. The major histocompatibility complex (MHC), referred to as the dog leukocyte antigen (DLA) system, plays a central role in antigen presentation and immune tolerance. Polymorphisms within DLA genes have been linked to autoimmune diseases such as diabetes mellitus and hypothyroidism, as well as differential vaccine response [1], and DLA haplotypes are associated with increased risk for autoimmune diseases in certain breeds [2,3]. Beyond the MHC, other genes involved in innate and adaptive immunity, such as Toll-like receptors and cytokine-related genes, have been implicated in breed-specific immune traits and inflammatory conditions [4]. Heritable disorders are related to different loci throughout the canine genome [5], often related to the canine breed. Regulation of these genetic changes can occur in different ways, but one of the most important is probably through epigenetic regulation. On the one hand, several studies have demonstrated the epigenetic regulation of canine mammary tumors [6] or the epigenetic reprogramming of macrophages that give rise to trained immunity [7]. Resistance to diseases in dogs could be associated with several epigenetic markers [8]. Knowing how these genes are regulated, and the possible involvement of epigenetic changes is essential to apply this knowledge to both veterinary medicine and human medicine, since dogs offer a unique model for dissecting the genetic architecture of immune regulation due to their breed structure and reduced genetic diversity within breeds, which enhances the power of genetic studies. In both humans and dogs, the immune system relies on coordinated actions of innate and adaptive cells, including dendritic cells, macrophages, neutrophils, NK cells, and T and B lymphocytes. Antigen presentation is mediated by MHC class I and II molecules that are encoded on chromosome 6 in humans (HLA-A, B, C, DR, DQ, and DP loci) and on chromosome 12 in dogs (DLA-A, -B, -DRB1, -DQA1, and -DQB1 loci) [9]. Canine DLA is less polymorphic and exhibits stronger haplotype linkage within breeds, leading to more uniform antigen presentation across individuals and breed-specific immune susceptibility. T-cell regulation in both species involves costimulatory and inhibitory checkpoints: CD28–CD80/86 interaction promotes activation, while CTLA-4 and PD-1/PD-L1 engagement restrains T-cell responses. These pathways are conserved in dogs, with increased CTLA-4 or PD-L1 expression observed in tumor-infiltrating lymphocytes and associated with poor prognosis in malignant canine tumors [10]. Functionally, canine and human NK cells share a similar transcriptomic profile—dogs more closely resemble humans than mice—while activated canine T cells show lower IFN-γ output than human T cells, despite macrophages demonstrating comparable or enhanced cytokine responses [11,12]. Finally, myeloid-derived suppressor cells and tumor-associated macrophages contribute to immunosuppressive tumor microenvironments by releasing IL-10, TGF-β, arginase, and VEGF, affecting both species and providing a rationale for cross-species evaluation of immune therapies [13].

Despite growing interest in canine immunogenetics, significant gaps remain in our understanding of the genetic regulation of immune responses in dogs. Compared to model organisms like mice and humans, the canine genome and its role in immune regulation are relatively under-characterized. For instance, while certain immune-related gene families such as the major histocompatibility complex (MHC), Toll-like receptors (TLRs), and cytokine genes have been partially mapped in dogs, their polymorphisms and functional implications in disease susceptibility and resistance are still poorly understood [2,14,15,16,17,18,19,20,21]. Moreover, breed-specific genetic bottlenecks complicate efforts to generalize findings across the species [22]. Limited transcriptomic and epigenetic data further restrict insight into regulatory mechanisms under various immunological challenges. These limitations hinder the development of breed-specific immunotherapies and vaccines and underscore the need for comprehensive multi-omics studies across diverse canine populations.

This review explores the current knowledge on the genetic regulation of immune responses in dogs, emphasizing key genes, signaling pathways, breed-specific variations, and implications for veterinary medicine.

## 2. Overview of the Canine Immune System Compared to Human

The immune system of mammals, including dogs and humans, consists of innate (physical barriers, phagocytes, such as macrophages, natural killer (NK) cells, and toll-like receptors (TLRs)) and adaptive immunity (T and B lymphocytes, antigen-presenting cells such as dendritic cells, and immunoglobulins). The coordination of these elements is under tight genetic control [23]. Dogs, like humans, have primary (thymus and bone marrow) and secondary (lymph nodes and spleen) lymphoid organs in charge of the maturation and proliferation of lymphocytes, T-cell subtypes with similar roles in antigen recognition and immune response coordination, and immunoglobulins. However, the two species present relevant differences, including the distribution and subtypes of immunoglobulins or the major histocompatibility complex (MHC), called dog leukocyte antigen (DLA) in dogs [24,25]. The last one, therefore, presents differences between canine breeds [26], further expanding the variety in the immune response and its regulation in this species.

Unlike the human immune system, which has four well-characterized immunoglobulin G (IgG) subclasses—IgG1, IgG2, IgG3, and IgG4—each with distinct roles in immune response, the canine immune system also has four IgG subclasses (IgG1–IgG4), but their specific functions are less well understood and show limited cross-reactivity with human antibodies. Additionally, while human major histocompatibility complex (MHC) genes are highly polymorphic and contribute to diverse immune recognition, the canine equivalent, known as dog leukocyte antigen (DLA), exhibits comparatively lower polymorphism [27]. Related to TLR, several species-specific signaling differences exist [28,29]. More of these differences between humans and dogs are related to genomic organization and gene regulation. For example, the location of MHC is different than DLA (MHC is located in human chromosome 6, whereas DLA is located in canine chromosome 12). Structural and genetic differences between these systems contribute to a more conserved immune response in dogs, particularly within individual breeds. This is due to the lower polymorphisms of DLA genes and the limited genetic diversity maintained through selective breeding, which leads to more uniform antigen recognition and, consequently, breed-specific immune responses and disease susceptibilities [30,31,32]. Similarly, the genomic sequences and expression patterns of TLRs differ between these two species. TLR5 and TLR9 show different sequences, whereas other genes encoding TLRs in dogs have functional orthologs, with differences in expression according to canine breed [33], which could affect susceptibility to infectious pathogens. Related to cytokines, although the genes encoding cytokines are more conserved between species, their expression profiles vary significantly due to promoter region variations and canine breed-specific polymorphisms. In fact, variants found in the gene encoding interleukin (IL)-10 have been associated with susceptibility of autoimmune hemolytic anemia in humans [34] but not in dogs [35].

The strong selection to which the canine species has been subjected in the generation of many breeds has caused significant differences in the immune response and its regulation depending on the breed. Concretely, some purebred dogs exhibit resistance or susceptibility to different diseases based on their genetic background or specific polymorphism fixed in this canine breed. DLA class II haplotypes have been associated with an increased risk of primary hypoadrenocorticism and symmetrical lupoid onychodystrophy in Border Collies [2], as well as with diabetes mellitus in Samoyeds, Tibetan Terriers, and Cairn Terriers [36], whereas polymorphisms in the *TLR4* and *TLR5* genes have been linked to inflammatory bowel disease in German Sheperd dogs [19]. In pathogen infections, polymorphism and haplotypes in several genes related to immune regulation have been associated with susceptibility to *Leishmania infantum* infection in Boxer dogs [20,21], whereas other genetic variants are related to infection resistance [37,38]. Regarding autoimmune diseases, haplotypes in DLA are related to canine rheumatoid arthritis in different breeds [39], and with canine systemic lupus erythematosus and retinal degeneration syndrome in the Nova Scotia Duck Tolling Retriever [5] and Dachshunds [40], respectively.

Therefore, identifying the genetic variants that are fixed and segregated in certain dog breeds is crucial for understanding genetic regulation and its influence on the immune response.

## 3. Toll-like Receptors (TLRs) and Innate Immune Gene Variability

**Figure 1:**
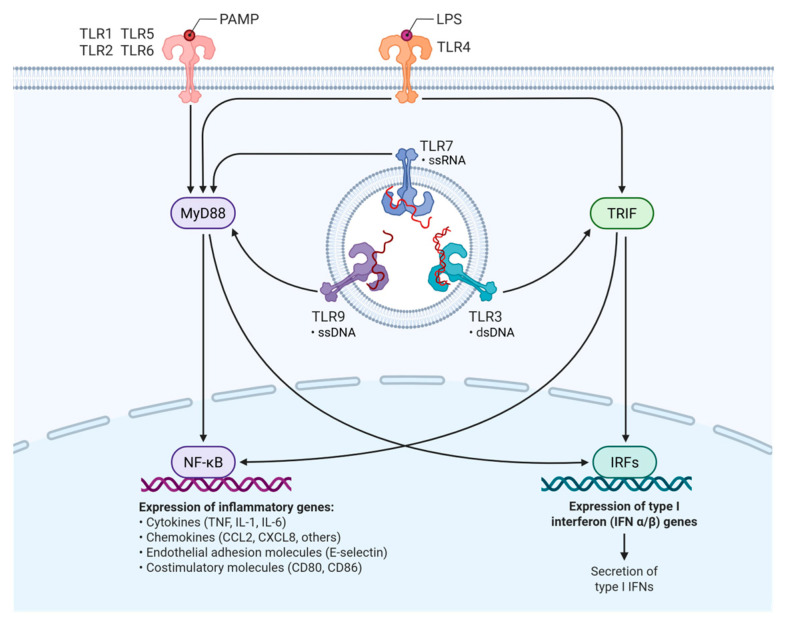
Toll-like receptor (TLR) signaling pathways involved in the innate immune response. TLRs recognize pathogen components and activate intracellular pathways leading to immune gene expression. TRL-1, -2, -5, and -6 are present on the surface and recognize bacterial components, whereas TLR-3, -7, and -9 are in the endosomes and recognized nucleic acids. NF-kB pathway leads to the production of inflammatory cytokines (TNF, IL-1, IL6), chemokines (CCL2 and CSCL8), and other immune-stimulating molecules, such as CD80 or CD86. IRF pathway stimulates the transcription of type I interferon genes, key in antiviral immunity [44].

Toll-like receptors (TLRs) are components of the innate immune response, acting as sensors of pathogen-associated molecular patterns (PAMPs) and endogenous damage signals (DAMPs) [41]. When one of the receptors recognizes either of these two groups of molecules, receptor dimerization and the activation of two intracellular signaling pathways are triggered: the MyD88 (myeloid differentiation primary response 88)-dependent pathway and the TRIF (TIR-domain-containing adapter-inducing interferon-β)-dependent pathway, which trigger the activation of the transcription factors NF-κB and IRF3. The activation of these transcription factors leads to an increase in the expression of certain genes that lead to the synthesis of inflammatory cytokines and type 1 interferon (IFN type 1) (Figure 1) [42,43].

TLRs are classified according to their cellular location and ligands. TLR-1, TLR-2, TLR-4, TLR-5, TLR-6, TLR-10, and TLR-11 receptors are expressed on the plasma membrane and primarily act as sensors of microbial surface components such as lipoproteins, lipopolysaccharides, and bacterial flagellar proteins like flagellin. In contrast, TLR-3, TLR-7, TLR-8, and TLR-9 receptors are located intracellularly in endosomes and lysosomes [42]. Their ligands are primarily viral or bacterial nucleic acids [45,46]. The TLR-10 receptor remains largely unknown today. However, a study confirmed the expression of this receptor in the lungs of dogs using Western Blot and immunohistochemistry, specifically in the bronchial subepithelium, the subendothelium of blood vessels, and the alveolar septa. Unlike other receptors, its specific ligand remains unknown. However, the localization of the TLR-10 receptor in specific regions of the lung highlights the possibility that this receptor plays a relevant role in the detection of respiratory pathogens [47]. In this species, the TLR-2 receptor forms heterodimers with TLR-1 and TLR-6 to recognize bacterial lipopeptides [48].

The TLRs are related to progression, resistance, and susceptibility to different canine diseases (Table 1). Different studies have also demonstrated the involvement and increased expression of the TLR-2 and TLR-4 receptors in *Leptospira* spp. infections, contributing to the production of inflammatory cytokines such as IL-1β by polymorphonuclear and peripheral mononuclear cells [49,50]. Also, in canine enteric infections caused by flagellated bacteria such as *Salmonella enteritidis*, the TLR-5 receptor responds to bacterial flagellin by triggering increased expression and the synthesis of cytokines IL-1β and IL-8 by peripheral mononuclear cells (PBMCs) [51]. Related to canine leishmaniasis, diseases caused by *Leishmania* spp. infection, several TLRs have been identified, among which TLR-2, TLR-4, and TLR-9 stand out, which are involved in the detection of parasite-derived components and the subsequent activation of the immune response. Specifically, alterations in the expression of these receptors in different regions have been observed during infection, decreasing the transcription of TLR-3, TLR-4, and TLR-9 when the disease progresses. In the spleen, a reduction in TLR-4 transcription has been detected in infected groups compared to uninfected individuals, while in the skin during the earliest stages of infection, an increase in TLR-9 transcription was revealed [52]. An increase in *TLR2* and *TLR4* gene expression has been observed in the earliest stages of the disease when there are practically no clinical signs. However, in more severe clinical stages, the expression of these genes decreases significantly, so these receptors could play an important role in controlling the infection in the early stages [53]. In this way, Grano et al. (2018) analyzed the gene expression of these receptors, as well as the production of inflammatory cytokines in the context of canine visceral leishmaniasis infection, revealing differences in the expression of these receptors in organs such as the spleen and brain. Genes encoding TLR-9 and TLR-5 were found to be overexpressed in the spleen, so they could play an important role in the innate immune response in leishmaniasis. Additionally, marked overexpression of the TLR-4 receptor was identified in regions such as the choroid plexus and leptomeninges, suggesting that this receptor may play an important role in the neuropathogenesis of the disease and may possibly be activated by other molecules, such as those associated with endogenous tissue damage (DAMPs). However, this result was observed in a small subpopulation and was not directly related to the parasitic load or clinical stage of the disease [54].

TLRs have also been implicated in the resistance mechanisms against canine leishmaniasis exhibited by certain breeds, such as the Ibizan Hound. Specifically, in this breed, it has been observed that, after TLR-2 stimulation with the agonist Pam3CSK4, either alone or in combination with the *Leishmania infantum* antigen, the synthesis of TNF-α and IL-6 is markedly accentuated, promoting the development of a Th1 immune response with the subsequent synthesis of IFN-γ [55,56]. In other parasitic diseases, such as demodicosis, changes in the expression of genes encoding TLR-2, TLR-4, and TLR-6 receptors have been observed. According to one study, *Demodex canis* modulates the gene expression of these receptors, which are key to innate immunity. Specifically, the parasite appears to promote the expression of TLR-2, which induces clinical signs of the disease and reduces the expression of TLR-4 and TLR-6, possibly with the aim of evading the host immune response. This imbalance in TLR expression contributes to the clinical manifestation of the disease and suggests a sophisticated immunomodulation mechanism by the mites [57].

A 2014 study by Cuscó et al. examined genetic variation in the innate immune response across seven dog breeds and two wolf lineages (Iberian and Russian), focusing on the genes encoding various TLRs. The results revealed that this genetic variability was not evenly distributed in the axonal regions of these genes. Notably, the plasma membrane-associated receptors TLR-5 and TLR-6 exhibited the highest number of non-synonymous single-nucleotide polymorphisms (SNPs), in contrast to intracellular receptors like TLR-3, which were highly conserved [58]. These differences reflect the distinct functional roles of the receptors, being intracellular TLRs, which detect viral and bacterial nucleic acids, subject to strong purifying (negative) selection to prevent harmful mutations, thereby maintaining their functional integrity [59]. Extracellular TLRs have evolved differently under less purifying selection pressure because they detect more variable molecules and possess a certain immunological redundancy. The study showed that Beagles and Russian-lineage wolves were the groups with the greatest allelic variations, while Iberian wolves, Boxers, and Yorkshire Terriers showed a lower number of segregating SNPs. Some of these polymorphisms were shared across breeds and lineages, such as TLR-4, in which the German Shepherd shared polymorphisms with Russian-lineage wolves. These polymorphisms alter the amino acid sequence of proteins, potentially implicating them in the innate immune response. Several SNPs were identified in the TLR-4 and TLR-5 genes in the German Shepherd breed that are associated with inflammatory bowel disease (IBD). The TLR-5 SNP G22A is associated with increased susceptibility to the disease, while the C100T and T1844C SNPs are more closely associated with a potential protective effect. Also, according to this latest study, the A1571T and G1807A SNPs, in combination with the TLR-5 SNP G22A, showed a significant association with inflammatory bowel disease (IBD) [19,58].

The TLRs appear to influence the pathogenesis of inflammatory colorectal polyps in miniature dachshunds. Exposing biopsies from affected and healthy dogs to ligands for the TLR-2, TLR-4, and TLR-9 receptors revealed an overexpression of proinflammatory cytokines compared to healthy individuals. This hyperreactivity to TLR ligands in affected individuals could be involved in the chronic colitis characteristic of dogs with ICRPs (inflammatory colorectal polyps) [60].

A recent study published in 2025 highlights the significant role of TLRs in the pathogenesis of atopic dermatitis [61]. In affected individuals, these receptors display dysfunction or hyporeactivity, triggering a reduction in the production of antimicrobial peptides, promoting the colonization of bacteria such as *Staphylococcus aureus*. In chronic stages, persistent TLR-2 activation drives a Th1 response with increased levels of IFN-γ and IL-12, contributing to the chronicity of the disease [61,62,63]. Multiple SNPs in the *TLR1*, *TLR2*, *TLR4*, *TLR6*, *TLR9*, and *TLR10* genes have been implicated in this disease. Notably, the *TLR2 R753Q* polymorphism is associated with reduced IFN-γ, increased IL-4 and IgE, and is involved in the development of an exaggerated allergic Th2 immune response [61,64,65]. 

This variant also leads to decreased IL-8 production in monocytes and increased IL-12 in macrophages, provoking chronic inflammation [61,66,67]. The role of TLR-9 SNPs remains unclear, so increased TLR-9 expression at birth appears protective [68]. And TLR-9 ligand exposure has shown therapeutic benefits [61,69,70,71]. Conversely, some promoter region polymorphisms enhance transcription and are linked to higher atopic dermatitis risk [61,72,73]. This disease is also related to overexpression of the *CD36* gene, which, combined with *TLR2 R753Q* polymorphism, may impair immune responses and increase susceptibility to secondary infections [74]. Although most studies focus on humans, these findings are relevant to veterinary medicine due to the similarities between canine and human atopic dermatitis [75].

**Table 1:** TLRs in specific canine diseases, including the key findings of these TLRs.

Diseases	Involved TLRs	Key Findings	References
*Leptospira* spp. infection	TLR-2, TLR-4	Increase IL-1β production	[37,38]
*Salmonella enteriditis*	TLR-5	Recognizes flagellin, increase IL-1β and IL-8 production	[39]
Canine leishmaniosis	TLR-2, -4, -5, and -9	Stage-specific expression. TLR-4 increases in early stages and decreases in late stages	[40,41,42]
Demodicosis	TLR-2, -4, and -6	Parasite modulates expression to evade immune response	[45]
Inflammatory bowel diseases (IBD)	TLR-4 and -5	SNPs in TLR-5: G22A increases the risk and C100T and T1844C with protective effect	[23,46]
Inflammatory colorectal polyps (ICRP)	TLR-2, -4, and -9	Increases the cytokine production after ligand exposure	[48]
Atopic dermatitis	TLR-1, -2, -4, -6, -9, and -10	SNP in TLR-2 R753Q, decreases IFN-γ and increases IL-4 levels	[49,50,51,52,53,54,55,60,61,62,63]
Canine lupus (DLE)	TLR-4	Overexpressed in skin; potential therapeutic target	[65]
Canine distemper (*Lycaon pictus*)	Multiple TLRs	Polymorphisms in TLR genes related to stronger immunity	[64]

## 4. Cytokine Genes and Immune Modulation

Cytokines play a key role in regulating the immune system by participating in processes such as the differentiation, proliferation, and activation of immune cells [76]. Among the most relevant cytokines in the immune response to pathogens and in vaccine efficacy are interleukin-2 (IL-2), interleukin-6 (IL-6), tumor necrosis factor alpha (TNF-α), and interferon gamma (IFN-γ) [21,77].

In canids, cytokine gene expression reflects considerable variability, largely influenced by the genetic background of different dog breeds. Various dog breeds display different transcriptional profiles in response to infectious agents or vaccines [20,78]. For example, the Ibizan Hound breed has specific haplotypes in the *IL6* and *IFNG* genes that are associated with elevated levels of IFN-γ, IL-2, and IL-18, which could confer greater resistance to diseases such as canine leishmaniasis [79]. One of the primary causes of this variability is the presence of single-nucleotide polymorphisms (SNPs) located in the promoter regions of these cytokine genes. These polymorphisms can alter the affinity of transcription factors and, consequently, transcription levels, significantly influencing the immune system [80].

In the context of bacterial and parasitic infections, Faria et al. (2011) evaluated the expression of cytokines such as TNF-α, IL-10, and interferon gamma (IFN-γ) in spleen cells and leukocytes during the acute phase of canine ehrlichiosis. The results showed an increase in TNF-α expression 18 days after inoculation, while after doxycycline administration, the levels of this cytokine were significantly reduced. These results suggest that TNF-α plays an important immunological role in the acute phase of the disease and that treatment with doxycycline reduces its levels, likely due to a decreased bacterial load [81]. Similarly, in puppies immunized with recombinant proteins against the nematode *Toxocara canis*, there were no significant differences with the control groups in terms of cytokine gene expression. However, a trend toward a multimodal immune response was observed, involving the activation of Th1 (IFN-γ-mediated), Th2 (IL-5-mediated), and Th17 (IL-17-mediated) pathways [82,83]. More recently, it has been demonstrated that coinfection with *Leishmania infantum* and *Leishmania tarentolae* modulates cytokine gene expression. Specifically, a decrease in the expression of the cytokines IL-4 and IL-6 and an increase in IL-12 were detected, suggesting an immune response oriented towards a protective Th1 immune profile [84].

Regarding the genetic regulation of cytokine expression, small non-coding RNA molecules (miRNA) have been shown to play a central role in the post-transcriptional regulation of cytokine-encoding genes [85]. In this context, the results of Rebech et al. (2023) showed a higher expression of miR-148a in the splenic leukocytes of animals infected by *L. infantum* compared to healthy individuals. Moreover, the in vitro results using a miR-148a mimetic molecule demonstrated a significant decrease in cytokines, such as TNF-α, IL-6, and IL-12 [86]. These proinflammatory cytokines are largely responsible for the development of a protective Th1 immune response that allows the destruction of *L. infantum* amastigotes [87]. These findings suggest that increased miR-148a expression may represent a parasite-mediated strategy to evade a protective immune response, thereby creating a more favorable environment for its persistence.

However, cytokines and their genetic regulation are not only crucial in the control of infections but also play a central role in the regulation of non-infectious pathologies, such as immune-mediated diseases, periodontal conditions, and otitis. The pathogenesis and progression of these disorders are significantly influenced by cytokine signaling. For example, canine atopic dermatitis is a chronic, genetically predisposed inflammatory skin disease characterized by a predominant T-cell-mediated immune response and sensitization to environmental allergens, often associated with impaired skin barrier dysfunction [88]. Schlotter et al. (2011) investigated the expression of cytokines and transcription factors in skin biopsies from dogs with and without atopic dermatitis lesions, as well as from healthy controls. The study revealed an elevated expression of interleukin-13 (IL-13) and suppressor of cytokine signaling 3 (SOCS3), both of which are indicative of the Th2 immune response [89]. Interestingly, the study also reported a significant increase in the expression of the transcription factor STAT4, typically associated with a Th1 immune response [90]. In contrast, the expression of GATA-3 (a transcription factor that suppresses Th1-related gene expression and enhances the production of Th2 cytokines) was reduced in lesional skin [89,91]. Additionally, IL-10, an immunosuppressive cytokine secreted by regulatory T cells, was upregulated in both lesional and non-lesional skin, potentially as a compensatory mechanism to counteract chronic inflammation [89,92]. Moreover, decreased expression of IL-12p40 in lesional skin suggests impaired Th1-mediated immunity and points to a dysfunction in the signaling pathway responsible for activating this arm of the immune response [93]. A recent study by Yoon and Park evaluated cytokine expression in the cerumen of dogs with otitis externa. Significant overexpression of IL-8 was reported in cases of Malassezia ceruminous otitis and bacterial suppurative otitis, reflecting a positive correlation with the degree of microbial burden. Interleukins such as IL-6 and IL-1β have also been identified as candidates for biomarkers of purulent inflammation in the ear canals in dogs with otitis externa, as both are significantly increased in dogs with bacterial suppurative otitis externa [94].

The immune system, along with genetics, plays a considerable role in the pathogenesis of diseases such as chronic canine enteropathy (CID) [95,96]. In this context, interleukins such as IL-25, IL-33, and thymic stromal lymphopoietin (TSLP) have been identified as key mediators of the Th2 immune response and proper intestinal homeostasis in humans [97]. In dogs, a study evaluated the expression of IL25, IL23, and TSLP genes in the duodenal and colonic mucosa in chronic enteropathy, showing a significant reduction in mRNAIL-33 levels in the duodenum in those dogs with diet-responsive enteropathy compared to healthy dogs. These results observed in dogs differ from those observed in humans with IBD, where IL-33 is overexpressed [98]. Furthermore, this decrease was not observed in dogs with antibiotic-responsive enteropathy or inflammatory bowel disease (IBD). Dysfunction in IL-33 levels can promote chronic inflammatory responses because this cytokine, in addition to acting as an “alarm” by activating immune system cells, also intervenes in the expansion and function of regulatory T lymphocytes [99,100].

A genome-wide association study (GWAS) conducted in the German Shepherd breed identified several polymorphisms significantly associated with canine inflammatory bowel disease (IBD). Some of these polymorphisms are located within or near genes that encode or regulate cytokines characteristic of the Th2 immune response, including IL-4, IL-5, IL-13, thymic stromal lymphopoietin (TSLP), and IL-33 [101], suggesting that genetic susceptibility to IBD in this breed may be linked to the expression of specific cytokines that contribute to dysregulated immune responses. These results align with observations in human ulcerative colitis, where the overexpression of cytokines such as IL-33 and TSLP has been documented, both of which are associated with an exaggerated Th2 immune response [15]. Although a polarization toward a Th2 or Th1 immune profile has not been established in canine IBD, the GWAS findings indicate the presence of polymorphisms that may indirectly influence the production of Th2-associated cytokines. In the context of other immune-mediated diseases in dogs, such as periodontal disease, seven novel variants in the *IL10* gene have been identified. These variants significantly affect the binding affinity of transcription factors to the gene’s promoter region, thereby modifying the expression levels of this cytokine. Although cytokine IL-10 levels were not directly quantified, specific *IL10* gene haplotypes were overrepresented in affected individuals, suggesting a possible regulatory impact [102]. Moreover, in canine necrotizing granulomatous meningoencephalitis, cytokine expression patterns linked to Th1, Th2, and Th17 responses were detected, indicating the involvement of a multimodal immune response in the disease pathogenesis [103].

Cytokines also play an essential role in determining the tumor microenvironment in situations where neoplasia is present. In this context, the study by Bujak et al. (2020) reported elevated IL17 expression in canine metastatic malignant mammary tumors [104]. Interestingly, the increase in IL-17 levels in tumor tissues was not associated with the expression of the transcription factor RORyt, which is essential for the differentiation of Th17 lymphocytes. This finding suggests that the IL-17 in these tumors may not originate from Th17 cells, but rather from tumor-associated macrophages (TAMs) or the tumor cells themselves. Increased expression of the chemokine receptors CXCR3 and CCR2-markers, typically associated with Th1 and Th2 immune responses, was also observed in malignant and metastatic tumors [103,104,105]. Similarly, the elevated expression of IL-12p40, a subunit involved in the formation of both IL-12 and IL-23, has been linked to the metastatic phenotype in these tumors [104]. In the case of canine histiocytomas, spontaneous tumor regression has been associated with progressive infiltration by lymphocytes (CD4+, CD8+, CD3+) and myelocytic or histiocytic cells. This cellular infiltration is accompanied by robust expression of Th-1-associated cytokines, including IL-2, TNF-α, IFN-γ, and the inducible nitric oxide synthase (iNOS) enzyme, all of which contribute to effective anti-tumor immune responses and tumor clearance [106].

## 5. Breed-Specific Immune Profiles and Heritable Disorders

Selective breeding has led to the emergence of distinct canine populations exhibiting diverse immune phenotypes. Genetic regulation of immune responses in dogs varies considerably among breeds and is influenced by selective breeding practices, reduced genetic diversity, and breed-specific dog leukocyte antigen (DLA) alleles. These factors contribute to both increased susceptibility and resistance to a variety of immune-mediated and immunodeficient conditions [107]. For example, breeds such as the Boxer, German Shepherd, and Labrador Retriever exhibit differential susceptibility to specific immune-mediated or infectious diseases, often linked to genetic variations in immune-related genes. Other breeds are predisposed to different diseases due to alterations in immune-related genes. Cavalier King Charles Spaniels demonstrated immunoglobulin deficiencies associated with susceptibility to *Pneumocystis pneumonia* infection [108], while Beagles show a tendency toward IgA deficiency, potentially predisposing them to chronic infections and autoimmune disorders [107]. Similarly, Doberman Pinschers are predisposed to hypothyroid disease [109] and Giant Schnauzers to autoimmune lymphocytic thyroiditis [110], likely related to polymorphisms in the DLA system present in both breeds. Single-nucleotide polymorphisms (SNPs) in key cytokine genes involved in Th1 and Th2 immune responses, such as IL-4 and IL-10, have been associated with diabetes mellitus in the Collie, Cairn Terrier, Schnauzer, and Cavalier King Charles Spaniel [111].

Wilbe and Andersson (2012) investigated the relationship between MHC class II genes, specifically the canine leukocyte antigen (DLA) and the genetic risk of developing lupus-like autoimmune diseases in dogs of the Nova Scotia Duck Tolling Retriever (NSDTR) breed. They identified a significant genetic association between certain MHC class II haplotypes and an autoimmune disease called immune-mediated rheumatic disease (IMRD), especially in dogs with positive antinuclear antibodies [112]. Haplotype 2 (DLA-DRB100601-DQA1005011-DQB1*02001) was found more frequently in dogs affected by IMRD than in healthy dogs. Dogs homozygous for this haplotype had a much higher risk of developing the disease. No clear association was found between these haplotypes and another related immune-mediated disease, steroid-responsive meningitis–arthritis (MSAR).

The NSDTR exemplifies a breed with a narrow genetic base and notable predisposition to several autoimmune disorders. This disease went through a bottleneck due to a canine distemper virus outbreak [113]. Among the most relevant conditions are systemic lupus erythematosus (SLE) and immune-mediated rheumatic disease (IMRD), both characterized by systemic inflammation and autoantibody production [108]. IgA deficiency has been detected in a significant percentage of NSDTRs and may predispose affected dogs to mucosal infections and other immune disorders [114]. These findings underscore the critical role of genetic regulation, particularly within the DLA class II region, in immune dysregulation in this breed. Another breed significantly affected by multiple autoimmune disorders is the American Akita. A strong genetic association has been documented between the DQA1*00201 allele and Uveodermatologic (Vogt–Koyanagi–Harada-like) syndrome, conferring a high relative risk (RR = 15.3) for this condition. Other prevalent autoimmune disorders in this breed include hypothyroidism (Hashimoto type), sebaceous adenitis, pemphigus foliaceus, and myasthenia gravis [115]. These associations illustrate how reduced DLA diversity due to inbreeding and the presence of risk alleles contribute to disease susceptibility. Severe combined immunodeficiency (SCID), a hereditary condition characterized by the inability to mount an antigen-specific immune response, has been described across several species, including humans, mice, horses, and dogs. In Jack Russell Terriers, a genetic predisposition to SCID by a mutation in the DNA-dependent protein kinase catalytic subunit (*DNA-PKcs*) gene exists, which is essential for DNA repair and lymphocyte development. In contrast, Cardigan Welsh Corgis and Basset Hounds develop SCID due to mutations in the gene encoding the common gamma (γ) chain, a shared component of interleukin receptors (IL-2, IL-4, IL-7, IL-9, IL-15, and IL-21). The specific mutation within these genes influences the phenotype and severity of SCID among breeds [116]. Dermatomyositis (DMS) is an immune-mediated disease with a known genetic predisposition in humans and several canine breeds, primarily Shetland Sheepdogs and Collies, but also in Beauceron. Although the responsible genes have not yet been identified, it is hypothesized that the Working Kelpie’s lineage from the Collie may explain a hereditary predisposition in the Australian Kelpie to DMS [117]. The genetic basis of DMS in Collies and Shetland Sheepdogs appears complex, involving multiple loci, including the *PAN2* gene on chromosome 10, the *MAP3K7CL* gene on chromosome 31, and the DLA class II haplotype, DLA-DRB1*002:01. Combinations of risk alleles at these loci correlate with differing susceptibility levels to DMS; dogs homozygous for risk alleles at *PAN2* and *MAP3K7CL*, and carrying the DLA-DRB1*002:01 haplotype, exhibit a higher risk of developing the disease [2,118]. This information is summarized in Table 2.

The high prevalence of immune-mediated diseases in purebred dogs strongly correlates with limited genetic diversity and breed-specific selection pressures. As noted by Pedersen, inbreeding contributes to immune dysregulation, promoting either autoimmunity or immunodeficiency [107]. Moreover, breeds predisposed to one immune disorder often present others, suggesting shared genetic susceptibility. Characterizing breed-specific immune profiles has practical implications for breeding strategies, diagnostics approaches, and the development of targeted gene therapies. Furthermore, these findings reinforce the dog’s value as a translational model for studying immune regulation in mammals.

## 6. Epigenetics and Immune Gene Regulation

**Figure 2:**
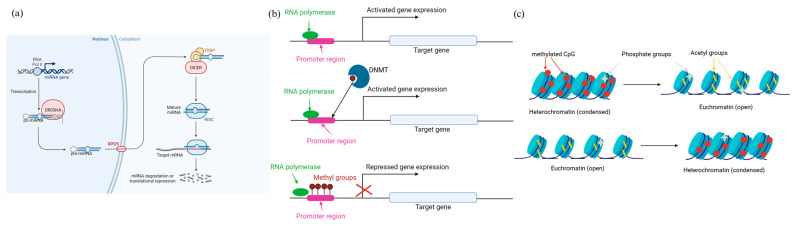
Epigenetic mechanisms of gene expression regulation: (**a**) MicroRNAs biogenesis and effect: In the nucleus, microRNAs (miRNAs) are transcribed by RNA polymerase II or III as primary transcripts (pri-miRNAs). These pri-miRNAs are processed by the enzyme Drosha and its cofactor DGCR8/Pasha, which cleaves them into precursor miRNAs (pre-miRNAs). The resulting pre-miRNAs are then actively transported to the cytoplasm by the nuclear export receptor Exportin 5 (XPO5) in a Ran-GTP–dependent manner. Once in the cytoplasm, the pre-miRNAs are further processed by the enzyme Dicer, producing short double-stranded mature miRNA molecules. These duplexes unwind, and one strand is incorporated into the RNA-induced silencing complex (RISC); (**b**) DNA methylation regulation: DNA methyltransferase (DNMT) enzymes add methyl groups to cytosine residues within CpG islands, typically located in gene promoter regions. This methylation prevents RNA polymerase from binding to the promoter, thereby repressing gene transcription and silencing gene expression; (**c**) histone modification regulation: Methyltransferases catalyze the addition of methyl groups to lysine and arginine residues on histones, typically promoting chromatin condensation and repressing gene expression. In contrast, acetyl transferases and deacetylases regulate the addition and removal of acetyl groups on lysine residues, leading to chromatin relaxation or condensation, respectively. Additionally, kinases and phosphatases modify histones by adding or removing phosphate groups at various sites, thereby influencing the chromatin structure and its accessibility for transcription [122].

Beyond DNA sequence variation, epigenetic mechanisms—such as non-coding RNAs, DNA methylation, and histone modifications—play roles in modulating immune gene expression (Figure 2). Although research in canine epigenetics is still emerging, preliminary studies suggest that environmental exposures and aging influence immune responses through epigenetic reprogramming. Regulation by non-coding RNAs (mainly micro-RNAs) occurs with the post-transcriptional silencing of target mRNAs through sequence-specific binding, leading to mRNA degradation or translational repression [119], whereas DNA methylation involves the addition of methyl groups to cytosine residues in CpG dinucleotides, typically leading to transcriptional repression by altering the chromatic structure or blocking transcription factor binding [120], and gene regulation by histone modifications refers the addition or removal of chemical groups (e.g., acetyl or methyl) to histone tails, altering the chromatin structure and the accessibility of DNA to transcriptional machinery [121].

Related to gene regulation by non-coding RNAs, microRNAs (miRNAs) are one of the most studied. These small non-coding RNAs regulate the posttranscription gene, affecting different biological processes. The studies in humans demonstrated that this regulation has great importance in the regulation of the immune system, observing the relevant role of miRNome in the regulation of B cells [123] and in inflammatory diseases [124]. However, the studies in dogs are scarce. One of the most studied is the role of miRNAs in immune gene regulation in *Leishmania* spp. infection. Since the host–parasite relationship and the activation of the Th1 or Th2 response are essential for the evolution of the infection, understanding the regulation of this immune response in this parasitic infection is of great interest. The activation of the Th1 response leads to the production of proinflammatory cytokines, such as IFN-gamma, among others, with a protective effect [125], while the activation of the Th2 response leads to the dissemination of the parasite and, therefore, to clinical signs that can be fatal [126]. In vitro studies with canine peripheral blood mononuclear cells (PBMC) have demonstrated that exposure to a parasite increases the expression of several miRNAs related to the regulation of the MAPK pathway, which is involved in immune response [127,128]. Even more, splenic leukocytes from dogs infected by and developing leishmaniosis, which express miRNAs related to immune system regulation, increase their production of IL-12 cytokine (proinflammatory cytokine related to Th1 response) when treated with an inhibitor of miR-21 [129]. In the same way, treatment with miR-148a inhibitors in splenic leukocytes from sick dogs decreases the iNOS levels and cytokines related to Th1, such as TNF-α, IL-6, and IL-12, but not in cells obtained from healthy dogs. These findings suggest a regulatory role of this miRNA in the immune response to this parasitic infection [86]. Similar results with other miRNAs have been observed in *Toxocara canis* infection, where the miRNAs expression profile changes during the parasitic infection stages [130]. The regulation of the canine immune system by microRNAs has not only been observed in parasitic infections but also in viral infections. This is the case of innate immune system inhibition from miR-485, miR-144, miR-133b, miR-4859-5p, miR-6902-3p, miR-7638, miR-1307-3p and miR-1346 in canine influenza virus infection [131], miR-526a in enterovirus infection [132] or cfa-miR-8908b and cfa.miR-146a in canid alphaherpesvirus 1 (CHV-1) [133]. Other miRNAs, such as miR-185, control the post-transcriptional regulation of genes encoding cytokines and their receptors, as it happens with the interleukin 17 receptor gene (*IL17R*) [134].

Although less studied, epigenetic mechanisms, such as DNA methylation and histone modifications, also appear to regulate the canine immune system in some way against some infections. In fact, lymphocytes of dogs with *Ehrlichia canis* infection show a decrease in histone H3 acetylation compared to the lymphocytes of healthy dogs [135]. In coronavirus and influenza viral infections, the genes related to antigen-presentation and the JAK-STAT pathway are downregulated by DNA methylation mechanisms, since certain viral proteins have the ability to bind to the cellular DNA methyltransferase enzyme DNMT3B, inhibiting its function [136,137]. DNA methylation appears to play a crucial role not only in the regulation of the immune system but also in the differentiation of T and B cells. An interesting study by Nam et al. (2023) conducted an exhaustive analysis of methylome in PBMC cells in canine mammary tumors, showing high methylation patterns in genes encoding proteins involved in the growth and differentiation of T and B cells, as well as genes related to immune cell proliferation. These findings indicate a highly relevant regulation of the canine immune system through DNA methylation [6].

## 7. Applications for Veterinary Medicine and Canine Health

Insight into the genetic regulation of immunity facilitates the development of breed-specific vaccination protocols, early detection methods for immune-related disorders, and targeted genetic screening initiatives. Genome-wide association studies (GWAS) and transcriptomic profiling are being increasingly used to identify immune regulatory *loci*, offering new avenues for precision veterinary care. The genetic regulation of the immune response in dogs has emerged as a key discipline in modern veterinary medicine. Advances in transcriptomics, comparative genomics, and functional analysis have made it possible to identify biomarkers, understand susceptibility to diseases, and improve diagnostic and therapeutic strategies.

Several studies have explored how the canine immune system responds to different virus infections, as well as how vaccines and therapies modulate this response. Gong et al. [138] developed a CRISPR/Cas9-based recombinant Canarypox vector vaccine expressing canine distemper virus (CDV) H and F proteins, which induces a strong humoral and cellular immune response, being safe and highly immunogenic. Another study tested a multiepitope vaccine containing immunodominant regions of CDV N, H, and F proteins in mice, showing good safety, neutralizing antibody production, and T-cell activation [139]. Biomarkers of immunoinflammation and oxidative stress were also identified in the serum and cerebrospinal fluid of dogs with distemper, offering tools for monitoring disease severity and progression [140]. Hama et al. (2025) identified in a murine model a non-canonical pathway of the RIG-I receptor activated by CaMKII during the acute phase of *Influenza A* virus infection [141]. This pathway induces IFN type I at low levels that paradoxically facilitate viral replication. The M3 inhibitor blocked this pathway and reduced the viral load, proinflammatory cytokines, and lethality, proposing itself as a novel antiviral strategy based on the inhibition of host proteins [141].

One of the most significant clinical applications is the use of immunotherapies, particularly immune checkpoint inhibitors (ICIs), in veterinary oncology. In malignant canine mammary tumors, immune checkpoint inhibitors (ICIs), such as PD-L1 and CTLA-4, have been associated with metastasis and poor prognosis, highlighting these pathways as potential therapeutic targets. However, response rates to ICIs in dogs remain modest (around 20%), lower than in human melanoma (40–60%) [142]. Canine sarcomas express multiple immunosuppressive markers, including PD-L1, CTLA-4, and LAG-3, indicating strong immune evasion capabilities. These profiles support combining ICIs with therapies like histotripsy [143]. In canine histiocytic sarcoma, transcriptomic analysis revealed three key genes: *PDCD1* (*PD-1*), overexpressed in tumors with higher T-cell infiltration; *SPP1* (osteopontin), linked to immunosuppression; and *TXNIP*, a suppressed tumor suppressor [144]. These patterns, also seen in human tumors, reinforce the use of dogs in comparative immunotherapy research. A meta-analysis across 19 canine cancers found variable expression of *PD-1*, *PD-L1*, *CTLA-4*, *LAG-3*, and *TIGIT*, highlighting the relevance of personalized approaches due to tumor heterogeneity [145]. In osteosarcoma, detailed molecular mapping identified 41 cell types, including immune cells as T and B lymphocytes, dendritic cells, and macrophages. Eight subtypes of the last ones and unique dendritic cells (mregCDs) with roles in adaptive immunity were observed. Regulatory and follicular helper T cells were also present, contributing to immune suppression. Follicular helper CD4 T cells (CD4fh) and regulatory T cells (Tregs) were identified, which play an important role in the suppression of the tumor immune response [145]. *PD-1*, *PD-L1*, and *PD-L2* expression were detected in untreated soft-tissue sarcomas, with reduced expression in the tumor areas affected by histotripsy, possibly enabling greater immune cell infiltration. Radiotherapy, with or without temozolomide, alters gene expression linked to the cell cycle, adhesion, and immune response, identifying survival-associated genes like *KRT76* and *ITGA8*, and the downregulation of *MMP13* and WNT5B [146]

These studies highlight the complexity and specificity of the canine immune response to different infectious agents. New technologies, such as recombinant vaccines, multiepitopic vaccines, and tissue transcriptomics, allow for the development of more effective and safer tools. In addition, immune modulation and inhibition of viral cell pathways represent innovative therapeutic approaches with great potential in veterinary and comparative medicine.

## 8. Conclusions

This review highlights the complexity and breed-specific nature of the canine immune system and its regulation, emphasizing the function, regulation, and control of diseases. Selective breeding has profoundly influenced the genetic regulation of immune responses in dogs, giving rise to distinct breed-specific immune phenotypes. These variations manifest as differential susceptibilities to infectious diseases, autoimmune disorders, and immunodeficiencies across breeds. Central to this diversity are breed-specific alleles in the dog leukocyte antigen (DLA) systems, which govern antigen presentation and immune recognition. Polymorphisms in key cytokine genes further modulate immune responses, contributing to breed-specific disease risks. Beyond DNA sequence variations, epigenetic regulation adds a critical layer of complexity to canine immune gene expression. Mechanisms such as microRNAs (miRNAs), DNA methylation, and histone modifications influence immune responses by altering transcriptional and post-transcriptional control. MiRNAs have emerged as key modulators of the immune system in dogs. DNA methylation, typically resulting in gene silencing, has been shown to downregulate genes that are essential for antigen presentation and cytokine signaling during infections, while histone modifications can either promote or repress chromatin accessibility, further shaping immune gene activity. These epigenetic changes often reflect environmental influences, aging, and disease states, highlighting their potential as diagnostic biomarkers and therapeutic targets in veterinary medicine.

The growing understanding of the genetic and epigenetic factors governing canine immunity is transforming veterinary practice by enabling the development of specific and more effective vaccines, early detection tools for immune disorders, and targeted immunotherapies. Genome-wide association studies (GWAS) and transcriptomic profiling have identified immune-related loci that inform precision veterinary care tailored to breed predispositions. Advances in vaccine technology, including recombinant and multiepitope vaccines, demonstrate improved safety and efficacy by eliciting robust humoral and cellular immunity against pathogens like the canine distemper virus. Furthermore, immunotherapies targeting immune checkpoints show promise in canine oncology, although response rates vary significantly between tumors and breeds. This variability underscores the necessity for personalized treatment strategies, supported by the molecular characterization of tumor immune microenvironments. Overall, dogs serve as valuable translational models for understanding immune regulation in mammals, bridging veterinary and human medicine. The genetic and epigenetic insights gained from canine studies not only advance animal health but also inform the broader immunological principles applicable to human diseases. Continued research into the interplay between inherited genetic variation, epigenetic modifications, and environmental factors promises to enhance disease prevention, diagnostics, and therapeutics. By integrating these molecular insights with clinical practice, veterinary medicine is moving toward a more precise and effective management of immune-mediated and infectious diseases, benefiting both canine populations and comparative immunology research.

## Figures and Tables

**Table 2:** Breed-specific resistance or susceptibility to selected canine diseases.

Diseases	Breed	Resistance/Susceptibility	References
*Pneumocystis pneumonia* infection	Cavalier King Charles Spaniels	Susceptibility due to immunoglobulin deficiencies	[103]
Chronic infections	Beagle	Susceptibility due to IgA deficiency	[102]
Hypothyroid disorders	Doberman Pinschers	Predisposition	[104]
Autoimmune lymphocytic thyroiditis	Giant Schnauzers	Predisposition	[105]
Diabetes mellitus	Collie, Cairn Terrier, Schnauzer, Cavalier King Charles Spaniel	Susceptibility associated with SNPs in cytokine genes	[106]
Lupus-like autoimmune disease	Nova Scotia Duck Tolling Retriever	Susceptibility related to haplotypes in DLA genes	[107]
Systemic lupus erythematosus	Nova Scotia Duck Tolling Retriever	Predisposition related to IgA deficiency	[110]
Uveodermatologic syndrome	American Akita	Risk due to allele of DLA	[111]
Severe combined immunodeficiency	Jack Russell Terriers	Predisposition due to SNP in kinase catalytic subunit	[112]
Severe combined immunodeficiency	Cardigan Welsh Corgis and Basset Hounds	Predisposition due to SNPs in gene-encoding interleukin receptors	[112]
Dermatomyositis	Collie, Shetland Sheepdog, and Beauceron	Susceptibility due to SNPs in different genes	[108]

## Data Availability

Not applicable.

## Abbreviations

The following abbreviations are used in this manuscript:

CCL	Chemokine (C-C motif) Ligand
CDV	Canine Distemper Virus
CD4fh	Follicular helper CD4 T
CHV-1	Canid Alphaherpesvirus 1
DLA	Dog Leukocyte Antigen
DAMP	Endogenous Damage Signal
DNMT	DNA Methyltransferase
IBD	Inflammatory Bowel Disease
ICI	Immune Checkpoint Inhibitor
ICRP	Inflammatory Colorectal Polyp
IFN	Interferon
Ig	Immunoglobulin
IL	Interleukin
IMRD	Immune-Mediated Rheumatic Disease
iNOS	Inducible Nitric Oxide Synthase
GWAS	Genome-Wide Association Studies
mregCD	Unique Dendritic Dell
miRNA	MicroRNA
MHC	Major Histocompatibility Complex
MyD88	Myeloid Differentiation Primary Response 88
NK	Natural Killer
PAMP	Pathogen-Associated Molecular Pattern
PBMCs	Peripheral Mononuclear Cells
SNP	Single Nucleotide Polymorphism
SOCS3	Suppressor of Cytokine Signaling 3
TAM	Tumor-Associated Macrophage
TRIF	TIR-Domain-Containing Adapter-Inducing Interferon-β
TLR	Toll-Like Receptor
TNF	Tumor Necrosis Factor
Tregs	Regulatory T cells
